# WO_3_/BiOBr S-Scheme Heterojunction Photocatalyst for Enhanced Photocatalytic CO_2_ Reduction

**DOI:** 10.3390/ma17133199

**Published:** 2024-06-30

**Authors:** Chen Li, Xingyu Lu, Liuyun Chen, Xinling Xie, Zuzeng Qin, Hongbing Ji, Tongming Su

**Affiliations:** 1 Guangxi Key Laboratory of Petrochemical Resource Processing and Process Intensification Technology, School of Chemistry and Chemical Engineering, Guangxi University, Nanning 530004, China; 15549366089@163.com (C.L.); 18736031369@163.com (X.L.); 18811313166@163.com (L.C.); xiexinling@126.com (X.X.); qinzuzeng@gxu.edu.cn (Z.Q.); jihb@mail.sysu.edu.cn (H.J.); 2 State Key Laboratory Breeding Base of Green-Chemical Synthesis Technology, Institute of Green Petroleum Processing and Light Hydrocarbon Conversion, College of Chemical Engineering, Zhejiang University of Technology, Hangzhou 310014, China

**Keywords:** WO_3_, BiOBr, heterojunction, photocatalytic, CO_2_

## Abstract

The photocatalytic CO_2_ reduction strategy driven by visible light is a practical way to solve the energy crisis. However, limited by the fast recombination of photogenerated electrons and holes in photocatalysts, photocatalytic efficiency is still low. Herein, a WO_3_/BiOBr S-scheme heterojunction was formed by combining WO_3_ with BiOBr, which facilitated the transfer and separation of photoinduced electrons and holes and enhanced the photocatalytic CO_2_ reaction. The optimized WO_3_/BiOBr heterostructures exhibited best activity for photocatalytic CO_2_ reduction without any sacrificial reagents, and the CO yield reached 17.14 μmol g^−1^ after reaction for 4 h, which was 1.56 times greater than that of BiOBr. The photocatalytic stability of WO_3_/BiOBr was also improved.

## 1. Introduction

The massive use of fossil fuel energy increases the amount of greenhouse gases, such as CO_2_, in the air, causing greenhouse effects, melting glaciers, and energy shortages [1,2,3]. Converting CO_2_ into chemical raw materials is a practical way to solve the environmental and energy crisis [4]. Among many technologies, photocatalytic reduction of CO_2_ to a chemical feedstock is a feasible way to achieve a green sustainable carbon cycle [5]. However, the low visible light absorption efficiency and recombination of the photoinduced electron-hole pairs in photocatalysts severely limit the efficiency of photocatalytic CO_2_ conversion [6,7]. Therefore, it is very significant to improve the photogenerated charge carriers separation in photocatalysts.

The construction of heterojunctions is considered to be beneficial for improving photocatalytic efficiency. Among various heterojunctions, S-scheme heterojunctions are widely studied due to their stronger redox ability [8]. In general, the S-scheme heterojunction includes an oxidation catalyst (OP) and a reduction catalyst (RP). When the RP is combined with the OP, the electrons will flow from the RP to the OP due to the higher Fermi level of the RP, resulting in the formation of an internal electric field at the interface. Under light irradiation, the electrons in the conduction band (CB) of OP combine with the holes in the valence band (VB) of RP due to the presence of an internal electric field, and the holes and electrons in the OP and RP can be used for oxidation or reduction reactions, respectively, thus promoting the separation of photogenerated electron-holes [9,10,11]. Therefore, the design of S-scheme heterojunctions with high charge carriers separation efficiency and strong redox capacity is a promising approach for improving photocatalytic efficiency.

Among many photocatalysts, bismuth-based photocatalysts have good photoelectric properties and are widely used in the fields of organic matter photodegradation and CO_2_ photoreduction [12,13]. Layered bismuth halides (BiOX, X = Cl, Br, and I) have been extensively studied due to their high photocatalytic performance [14]. BiOX consists of a bismuth–oxygen layer (Bi_2_O_2_)^2+^ and two alternatingly arranged Br layers. Such a layered structure can form an internal electric field to shorten the transmission distance of the photogenerated electrons, which is very favorable for photocatalytic reactions [15,16,17]. Notably, BiOBr has aroused wide concern due to its narrow band gap and suitable band structure. However, the rapid recombination of photoinduced charge carriers in BiOBr limits its application in photocatalytic CO_2_ reduction [18]. To improve the photocatalytic efficiency of BiOBr, numerous ways have been proposed, such as regulating oxygen vacancies [19], metal doping [20], and hybridization with various semiconductors [21,22,23]. The formation of an S-scheme heterojunction can be a feasible way to improve the photocatalytic efficiency of BiOBr [24,25,26]. The use of a suitable oxidized semiconductor combined with BiOBr to construct an S-scheme heterojunction can shorten the transfer distance of charge carriers, prolong the carrier lifetime, and enhance the redox ability. For instance, WO_3_ is widely used as the oxidizing photocatalyst due to its advantages of low cost, excellent photoelectric performance, and strong oxidation ability [27,28]. Moreover, WO_3_ has a suitable band structure and can form S-scheme heterojunctions with BiOBr, which is expected to enhance the performance of photocatalytic CO_2_ reduction.

In this work, WO_3_/BiOBr heterojunctions were prepared and used for photocatalytic CO_2_ reduction. Under visible light (λ ≥ 400 nm), the optimized WO_3_/BiOBr exhibited the best CO yield of 17.14 μmol g^−1^ irradiation for 4 h without the addition of sacrificial agents, which was 1.56 times higher than that of BiOBr. Combining the activity data and characterization results, the S-scheme charge transfer mechanism was demonstrated for WO_3_/BiOBr, which enhances the rapid transfer and separation of photoinduced charge carriers.

## 2. Experimental

### 2.1. Synthesis of the Photocatalyst

#### 2.1.1. Materials

Bi(NO_3_)_3_·5H_2_O was obtained from Guangdong Guanghua Technology Co., Ltd. (Shantou, China). Polyvinylpyrrolidone (PVP, K-30) was purchased from Shanghai McLean Biochemical Technology Co., Ltd. (Shanghai, China). KBr was purchased from Tianjin Guangfu Technology Development Co., Ltd. (Tianjin, China). WO_3_ was purchased from Tianjin Bodi Chemical Co., Ltd. (Tianjin, China). All the chemical agents involved in this work were analytically pure and were used without further purification.

#### 2.1.2. Preparation of BiOBr

First, 5 mmol of Bi(NO_3_)_3_·5H_2_O and 0.2 g of PVP were mixed in 40 mL deionized (DI) water and stirred for 60 min. At the same time, 5 mmol of KBr was dissolved in 40 mL of DI water and stirred for 60 min. After that, the above solution was mixed and stirred together, and the pH was adjusted to 6. The mixture was then stirred for 1 h and heated at 160 °C for 12 h. After cooling down, the mixture was washed with DI water several times and dried at 60 °C for 12 h in a vacuum drying oven.

#### 2.1.3. Preparation of WO_3_/BiOBr

First, 0.2 g of BiOBr was dispersed in 40 mL DI water for 30 min by ultrasonication, and then a certain amount of WO_3_ (3, 5, 10 wt%) was added to the above suspension and stirred at 60 °C for 10 h by water bath. The sample was then washed with DI water and dried by vacuum drying at 60 °C for 12 h. The samples with different mass ratios were named 3WO_3_/BiOBr, 5WO_3_/BiOBr, and 10WO_3_/BiOBr.

### 2.2. Photocatalytic CO_2_ Reduction

First, 30 mg photocatalyst was dispersed evenly in 1.5 mL DI water by ultrasonication, and then the photocatalyst was evenly spread on a quartz sheet and dried at 60 °C in a vacuum drying oven. For the photocatalytic CO_2_ reduction, the quartz sheet with the photocatalyst was placed at the bottom of the reactor. After that, the wet CO_2_ was injected into the reactor for 30 min, with a flow quantity of 40 mL min^−1^ to ensure that the air was completely removed. A 300 W xenon lamp (CEL-HXF300, Beijing China Education Au-light Co., Ltd., Beijing, China) was applied as the light source for the reaction and a 400 nm cutoff filter was used to filter out light below 400 nm. The light intensity of the light source was determined to be 178 mW cm^−2^ by using an optical power meter (CENP2000, Beijing China Education Au-light Co., Ltd., Beijing, China). The reaction temperature was maintained at 25 °C by using circulating cooling water. Every hour, 0.5 mL of gas product was detected on a GC-2030 gas chromatograph (Shimadzu, Kyoto, Japan).

### 2.3. Characterization

The XRD patterns were obtained on a Bruker D8 X-ray diffractometer (BRUKER AXS GMBH, Karlsruhe, Germany). Fourier transform infrared spectra and in situ infrared spectra were recorded on a Bruker Tensor II infrared spectrometer (Bruker, Karlsruhe, Germany). SEM images were obtained on a ZEISS GeminiSEM 300 instrument (ZEISS, Oberkochen, Germany). TEM images and elemental mapping images were recorded on a JEOL JEM-F200 transmission electron microscope (JEOL, Tokyo, Japan). XPS spectra were performed on a Thermo Scientific K-Alpha instrument (Thermo Fisher Scientific, Waltham, MA, USA). Ultraviolet-visible diffuse reflectance spectra were analyzed by a TU-19 spectrophotometer (PERSEE ANALYTICS, Beijing, China). N_2_ adsorption and desorption curves were obtained on a Tristar II physical adsorption instrument (Micromeritics, Norcross, GA, USA). Time-resolved fluorescence spectra (TRPL) were analyzed on an Edinburgh FLS 1000 spectrometer (Edinburgh Instruments, Livingston, UK).

### 2.4. Photoelectrochemical Measurements

A CHI 760E electrochemical workstation was used for the photoelectrochemical measurements (Shanghai Chenhua Instrument Co., Ltd., Shanghai, China). The test was carried out in a three-electrode system, with an Ag/AgCl electrode, a Pt electrode, and a photocatalyst electrode used as the reference electrode, the counter electrode, and the working electrode, respectively. In addition, 0.5 M Na_2_SO_4_ was used as the electrolyte. To prepare the working electrode, 20 mg of photocatalyst was added to 400 μL absolute ethanol and 20 μL of Nafion^®^ solution (Shanghai McLean Biochemical Technology Co., Ltd, Shanghai, China), and the mixture was ultrasonicated at 25 °C for 2 h. Then, 20 μL of the mixed solution was uniformly dropped on FTO conductive glass with a central area of 1 cm^2^ (2 cm × 2 cm) and the working electrode was dried naturally. The electrochemical impedance spectrum (EIS) was tested at an alternating amplitude of 5 mV and with a frequency from 0.01 to 1,000,000 Hz. The transient photocurrent response was tested with a 300 W xenon lamp (equipped with a 400 nm cutoff filter) as the light source. Mott–Schottky curves were obtained at frequencies of 1000, 1500, and 2000 Hz.

## 3. Results and Discussion

### 3.1. Structure and Morphology

**Figure 1:**
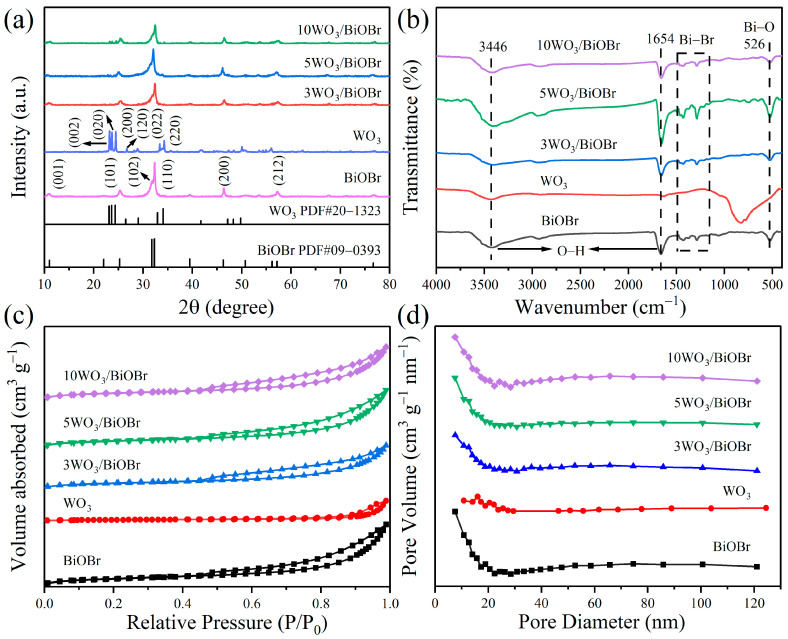
XRD patterns (**a**), FT–IR spectra (**b**), N_2_ adsorption–desorption isotherms (**c**), and pore size distributions (**d**) of BiOBr, WO_3_, and xWO_3_/BiOBr.

The crystalline phase and composition of BiOBr, WO_3_, and xWO_3_/BiOBr were measured by XRD. From Figure 1a, the peaks of WO_3_ are consistent with the triclinic phase WO_3_ (PDF#20−1323) [29]. In the XRD patterns of BiOBr, the diffraction peaks at 10.9°, 25.1°, 31.7°, 32.2°, 46.2°, and 57.1° are ascribed to the (001), (101), (102), (110), (200), and (212) planes of the tetragonal phase BiOBr, respectively (PDF#09−0393) [30]. Notably, from Figure 1a and Figure S1, enlarged regions from 20° to 30° in XRD patterns of xWO_3_/BiOBr are shown in Figure S1, with the peaks at 23.1°, 23.6°, 24.4°, and 26.8° ascribed to the (002), (020), (200), and (120) planes of WO_3_ (PDF#20−1323), and the peak at 25.1° is attributed to the (101) plane of BiOBr (PDF#09−0393). Both the peaks of WO_3_ and BiOBr can be found in the XRD patterns of xWO_3_/BiOBr, indicating that the xWO_3_/BiOBr composites were successfully prepared.

FT–IR spectra were used to further study the structure of the obtained samples. Figure 1b shows that the peaks at 526 cm^−1^ are assigned to the tensile vibration of the Bi–O bond [31,32], and the peaks located in the range of 1000~1500 cm^−1^ region are ascribed to the asymmetric and symmetric vibrations of the Bi–Br bond [33,34]. The peaks of WO_3_ at 826 cm^−1^ are attributed to the W–O bond [35]. In addition, the peaks at 1654 cm^−1^ are attributed to the bending vibrations of the adsorbed H_2_O on the surface of BiOBr, and the peaks at 3436 cm^−1^ correspond to the stretching vibrations of the adsorbed OH groups [36]. However, due to the low content of WO_3_, the peaks of WO_3_ cannot be seen from the FT–IR spectra of xWO_3_/BiOBr.

The specific surface area and average pore diameter of the samples were studied by N_2_ adsorption and desorption. Figure 1c shows that all the samples have the type IV isotherms and type H3 hysteresis loops, indicating that the samples are mesoporous material [37]. The specific surface areas of BiOBr, WO_3_, 3WO_3_/BiOBr, 5WO_3_/BiOBr, and 10WO_3_/BiOBr are 19.64, 3.54, 14.81, 17.96, and 17.15 m^2^ g^−1^ (Table S1), respectively, indicating that the specific surface area of xWO_3_/BiOBr composites is slightly lower than that of BiOBr but much higher than that of WO_3_. Among the xWO_3_/BiOBr composites, 5WO_3_/BiOBr exhibits the largest specific surface area and can provide more reaction sites for photocatalytic reactions [38].

**Figure 2:**
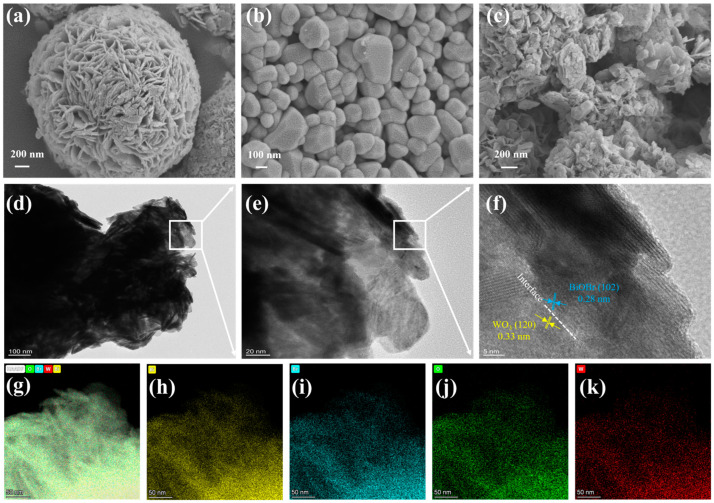
SEM images of BiOBr (**a**), WO_3_ (**b**), and 5WO_3_/BiOBr (**c**). TEM and HRTEM images (**d**–**f**), HAADF image (**g**), and corresponding EDX mapping profiles of Bi (**h**), Br (**i**), O (**j**), and W (**k**) of 5WO_3_/BiOBr.

Scanning electron microscopy (SEM) and transmission electron microscopy (TEM) were used to investigate the morphology and microstructure of the catalyst (Figure 2). BiOBr shows the nanoflower structure assembled from many nanosheets, while WO_3_ has a polyhedral structure. From the SEM images of 5WO_3_/BiOBr, we can see that the BiOBr nanoflower was dispersed and covered on the surface of WO_3_, which is beneficial for increasing the contact interface between WO_3_ and BiOBr. In addition, the SEM images of 3WO_3_/BiOBr and 10WO_3_/BiOBr are also shown in Figure S2. The microstructure of 5WO_3_/BiOBr was further investigated by HRTEM. Figure 2e and Figure 2f are the enlarged regions of the white boxes in Figure 2d and Figure 2e, respectively. As shown in Figure 2f, the lattice fringe spacing of 0.33 and 0.28 nm is attributed to the (120) planes of WO_3_ [39] and (102) planes of BiOBr [40], respectively. Notably, a tight contact interface between WO_3_ and BiOBr is clearly observed, indicating the formation of the WO_3_/BiOBr heterojunction. Moreover, the lattice fringe of WO_3_ can also be observed close to the lattice fringe of BiOBr, indicating the successful construction of the heterointerface between WO_3_ and BiOBr. In addition, according to the EDS elemental mapping of 5WO_3_/BiOBr (Figure 2g–k), the Bi, O, Br, and W elements are evenly distributed in the 5WO_3_/BiOBr composite. According to the EDX spectrum (Figure S3), the Bi, Br, O, and W elements exist in 5WO_3_/BiOBr and the mass fraction and atomic fraction of W are 2.23% and 1.31%. These results indicate the successful synthesis of 5WO_3_/BiOBr composites.

### 3.2. Surface Chemical State

The surface chemical state of the photocatalysts was investigated by XPS spectra. The XPS survey spectra of BiOBr, WO_3_, and 5WO_3_/BiOBr display that Bi, O, Br, and W elements are present in 5WO_3_/BiOBr, which demonstrates the successful synthesis of 5WO_3_/BiOBr (Figure S4). The enlarged spectral regions from 0 eV to 100 eV in the XPS survey spectra of BiOBr, WO_3_, and 5WO_3_/BiOBr are shown in Figure S4b. The peaks of Bi 5d, O 2s, W 4f_7/2_, and W 4f_5/2_ are found at 25.9, 25.9, 35.9, and 37.9 eV, respectively. Overtly, no WO_3_ signal was found in enlarged spectral regions from 0 eV to 100 eV of BiOBr. 

**Figure 3:**
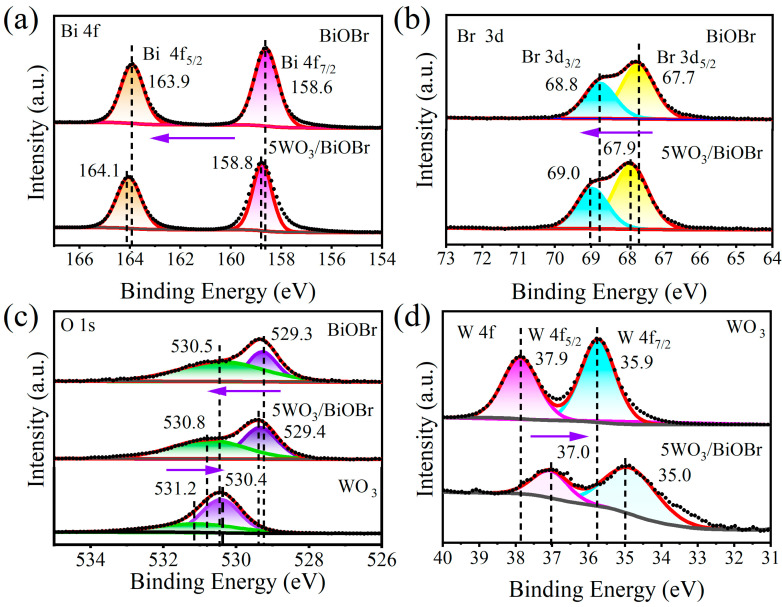
XPS spectra of Bi 4f (**a**), Br 3d (**b**), O 1 s (**c**), and W 4f (**d**) in BiOBr, WO_3_, and 5WO_3_/BiOBr.

Figure 3 shows the XPS spectra of Bi 4f, Br 3d, O 1s, and W 4f. As can be seen from the XPS spectra of BiOBr (Figure 3a), the two peaks at 158.6 and 163.9 eV are ascribed to Bi 4f_7/2_ and Bi 4f_5/2_ for Bi^3+^, respectively. Compared to BiOBr, the binding energies of Bi 4f_7/2_ and Bi 4f_5/2_ in 5WO_3_/BiOBr shifted to 158.8 and 164.1 eV, respectively [41,42]. From the Br 3d spectra of BiOBr and 5WO_3_/BiOBr (Figure 3b), two peaks at 68.8 and 67.7 eV are attributed to Br 3d_3/2_ and Br 3d_5/2_, respectively, indicating the presence of Br^−^ [43], while the binding energies of Br 3d_3/2_ and Br 3d_5/2_ in 5WO_3_/BiOBr shift to 69.0 and 67.9 eV, respectively. The shift of the Bi 4f_5/2_, Bi 4f_7/2_, Br 3d_3/2_, and Br 3d_5/2_ in 5WO_3_/BiOBr indicates that the electrons were migrated from BiOBr to WO_3_ after BiOBr was contacted with WO_3_.

As shown in the O 1s spectra of BiOBr, WO_3_, and 5WO_3_/BiOBr (Figure 3c), the binding energy of W–O is located at 530.4 and 531.2 eV for WO_3_ [44], the binding energy of Bi–O is located at 529.3 and 530.5 eV for BiOBr [45], and the peaks shift to 529.4 and 530.8 eV, respectively, in the O 1s XPS spectrum of 5WO_3_/BiOBr, indicating that the chemical environment was changed after the formation of the WO_3_/BiOBr heterojunction. In addition, two peaks located at 37.9 and 35.9 eV were found in the W 4f XPS spectrum of WO_3_, which correspond to W 4f_5/2_ and W 4f_7/2_ for W^6+^ [46], respectively (Figure 3d). In the XPS spectra of 5WO_3_/BiOBr, W 4f_5/2_ and W 4f_7/2_ shifted to 37.0 and 35.0 eV, respectively, indicating that the electron cloud density of the W in WO_3_ was increased. The above result indicates that the electrons in BiOBr can migrate to WO_3_ through the interface after WO_3_ was coupled with BiOBr, and a built-in electric field was constructed at the WO_3_/BiOBr interface [47,48].

### 3.3. Light Absorption Capacity and Band Structure

**Figure 4:**
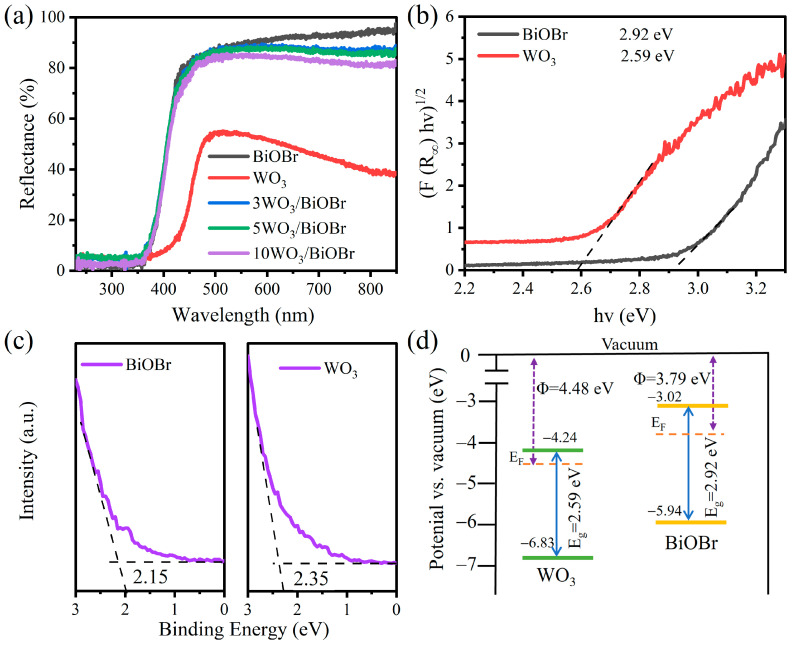
UV–Vis DRS of BiOBr, WO_3_, and xWO_3_/BiOBr (**a**), band gap of BiOBr and WO_3_ (**b**), and UPS spectra (**c**) and band structure (**d**) of WO_3_ and BiOBr.

The light absorption capacity and band structures of WO_3_, BiOBr, and 5WO_3_/BiOBr were analyzed by UV–Vis DRS and UPS spectroscopy (Figure 4). As shown in Figure 4a, WO_3_ shows obvious visible light absorption. However, no significant changes were observed for the light absorption capacity of BiOBr after the introduction of WO_3_. The band gaps of BiOBr and WO_3_ were determined to be 2.92 and 2.59 eV by the Kubelka-Munk method, respectively (Figure 4b), which is in accordance with the results in the literature [49,50].

UPS spectra were carried out to analyze the work function (Φ) and valence band (VB) of the catalyst (Figure 4c and Figure S5). As shown in the UPS spectra of BiOBr and WO_3_ (Figure S5), the secondary electron cutoff edges (E_cutoff_) of BiOBr and WO_3_ are 17.43 and 16.74 eV, respectively. As a result, the work functions of BiOBr and WO_3_ were calculated to be 3.79 and 4.48 eV, respectively, according to Equation S1 [51]. In addition, the value of the work function is equal to the difference value between the vacuum level (E_v_) and the Fermi level (E_f_). Herein, the value of the vacuum level is identified as 0 eV [52], and the Fermi levels of BiOBr and WO_3_ were calculated to be −3.79 and −4.48 eV relative to the vacuum level. In addition, as shown in Figure 4c, the valence band (VB) values of BiOBr and WO_3_ are 2.15 and 2.35 eV relative to the Fermi level, respectively. Therefore, the VB values of BiOBr and WO_3_ were calculated to be −5.94 and −6.83 eV (vs. vacuum), respectively [53]. Therefore, the conduction band (CB) values of BiOBr and WO_3_ were determined to be −3.02 and −4.24 eV (vs. vacuum), respectively [54]. As shown in the band structures of BiOBr and WO_3_ (Figure 4d), the electrons in BiOBr will transfer to WO_3_ due to the higher work function of WO_3_, and a built-in electric field can be formed at the WO_3_/BiOBr interface, which is in favor of enhancing the separation of photoinduced electron-hole pairs.

In addition, Mott–Schottky plots were obtained to analyze the flat-band potentials (E_fb_) of the samples. From Figure S6, the positive slopes of the Mott–Schottky plots indicate that both BiOBr and WO_3_ are n-type semiconductors [55]. The E_fb_ values of BiOBr and WO_3_ were determined to be −0.45 and −0.26 V (vs. Ag/AgCl, pH = 7), respectively. According to Equation (S2) [56], the E_fb_ values of BiOBr and WO_3_ were calculated to be 0.16 and 0.35 V (vs. NHE, pH = 0), respectively. In general, for n-type semiconduction materials, the flat band potential is approximately equal to the Fermi level [57]. Therefore, the E_f_ values of BiOBr and WO_3_ were 0.16 and 0.35 V (vs. NHE, pH = 0), indicating that the Fermi level of WO_3_ is lower than that of BiOBr. This confirms that the electrons in BiOBr can migrate to WO_3_ due to the higher Fermi level of BiOBr, which is in accordance with the UPS results.

### 3.4. Charge Transfer and Separation

**Figure 5:**
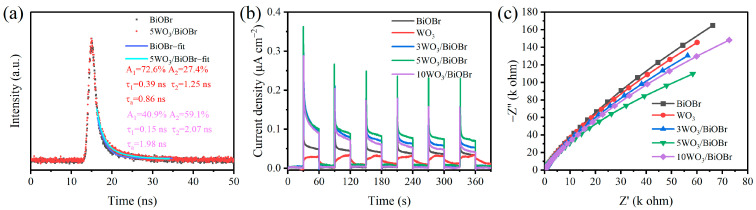
TRPL spectra of BiOBr and 5WO_3_/BiOBr (**a**), transient photocurrent density (**b**), and EIS Nyqui st plots (**c**) of BiOBr, WO_3_, and xWO_3_/BiOBr.

The charge transfer kinetics of BiOBr and 5WO_3_/BiOBr were analyzed by time-resolved photoluminescence (TRPL) spectra (Figure 5a). In general, the carrier lifetime is estimated by the photoluminescent decay time, and the average fluorescence lifetime can be calculated by Equation (1) [58,59], where A_1_ and A_2_ are pre-exponential factors and τ_1_ and τ_2_ represent the lifetimes of radiative and nonradiative transitions, respectively. Figure 5a shows that the average fluorescence lifetimes of BiOBr and 5WO_3_/BiOBr are 0.86 ns and 1.98 ns, respectively. The prolongation of the fluorescence lifetime in 5WO_3_/BiOBr indicates that the separation efficiency of photoinduced electrons and holes was enhanced after the WO_3_/BiOBr interface was formed [60].

(1) $$\tau_{a}=\frac{A_{1}\tau_{1}^{2}+A_{2}\tau_{2}^{2}}{A_{1}\tau_{1}+A_{2}\tau_{2}}$$

The transient photocurrent densities were also investigated under visible light (λ ≥ 400 nm) to analyze the transfer and separation of the photogenerated charge carriers. As displayed in Figure 5b, the transient photocurrent density of 5WO_3_/BiOBr is higher than that of other samples, indicating that the charge separation efficiency in 5WO_3_/BiOBr is higher. In addition, the EIS Nyquist plots of BiOBr, WO_3_, and xWO_3_/BiOBr were obtained to explore the transfer resistance of the electrons. In general, the arc radius of the Nyquist curve is proportional to the impedance of the photocatalyst [61]. As shown in Figure 5c, the radius of 5WO_3_/BiOBr is smaller than that of WO_3_, BiOBr, 3WO_3_/BiOBr, and 10WO_3_/BiOBr, indicating that the charge transfer resistance of 5WO_3_/BiOBr was reduced after BiOBr coupling with WO_3_. Among them, 5WO_3_/BiOBr exhibited the highest photocurrent density and the smallest impedance arc radius, indicating the most efficient separation of photogenerated electron-hole pairs and the fastest photogenerated charge transfer on 5WO_3_/BiOBr. Based on the above discussion, the formation of the WO_3_/BiOBr heterojunction is beneficial for improving the charge separation efficiency and improving the photocatalytic performance.

### 3.5. Photocatalytic CO_2_ Reduction

**Figure 6:**
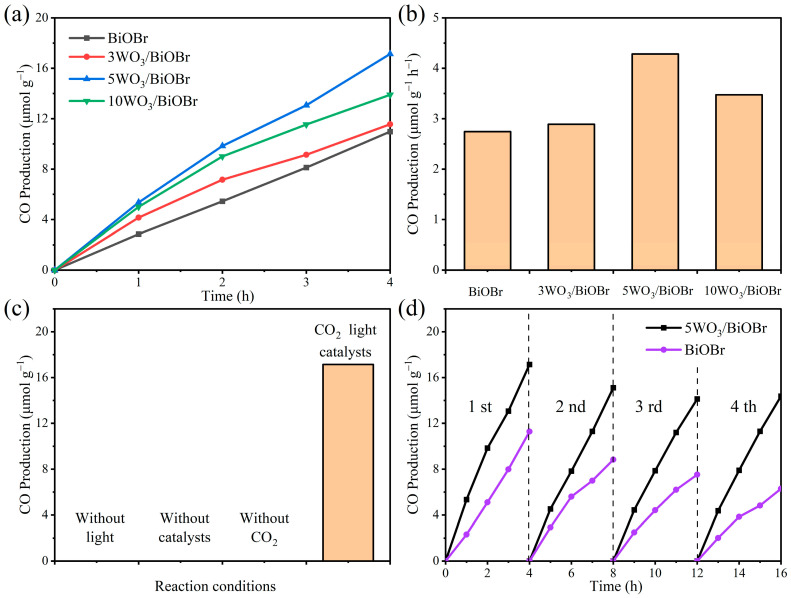
Time course of photocatalytic CO_2_ reduction over BiOBr and xWO_3_/BiOBr (**a**,**b**), photocatalytic CO_2_ reduction over 5WO_3_/BiOBr under different conditions (**c**), and cycle test of BiOBr and 5WO_3_/BiOBr photocatalytic CO_2_ reduction to CO (**d**).

The photocatalytic CO_2_ reduction activity test over BiOBr and xWO_3_/BiOBr was carried out under visible light (Figure 6). Figure 6a,b show that the photocatalytic CO_2_ reduction activity of xWO_3_/BiOBr was greater than BiOBr. Moreover, the CO production over xWO_3_/BiOBr first increased and then decreased with the increase of the WO_3_ amount. Among them, 5WO_3_/BiOBr exhibited the best CO production of 17.14 μmol g^−1^ after 4 h reaction, which is 1.56 times greater than that of BiOBr (10.96 μmol g^−1^). The higher photocatalytic performance of 5WO_3_/BiOBr can be ascribed to the enhanced separation of photogenerated charge carriers at the WO_3_/BiOBr interface. To demonstrate the advantages of 5WO_3_/BiOBr, the photocatalytic CO_2_ reduction performance of the BiOBr-based photocatalysts are listed in Table S2. Compared with the other photocatalysts shown in Table S2, 5WO_3_/BiOBr presented satisfactory photocatalytic CO_2_ reduction activity. Among them, 5WO_3_/BiOBr exhibited the highest photocurrent density and the smallest impedance arc radius, indicating the most efficient separation of photogenerated electron-hole pairs and the fastest photogenerated charge transfer on 5WO_3_/BiOBr. Therefore, the CO production rate of 5WO_3_/BiOBr is higher than that of 3WO_3_/BiOBr and 10WO_3_/BiOBr. In addition, when the amount of WO_3_ is 3 wt%, a small number of heterojunctions cannot effectively promote the separation of photogenerated electrons and holes. However, when the amount of WO_3_ is 5 wt%, excessive WO_3_ will limit the light absorption capacity of BiOBr and cover the active site of CO_2_ reduction of BiOBr. 

Controlled experiments were conducted to confirm the influence factors and C source of the product. From Figure 6c, when the photocatalytic reaction was carried out without light, a catalyst, or CO_2_, no CO product was detected, indicating that light, catalyst, and CO_2_ are the necessary conditions for photocatalytic CO_2_ reduction reaction. The stability of BiOBr and 5WO_3_/BiOBr was also studied. As shown in Figure 6d, after four cycles, the CO production over BiOBr and 5WO_3_/BiOBr decreased by 44.1% and 16.2%, respectively, indicating that the combination of WO_3_ with BiOBr can improve the stability of BiOBr during the photocatalytic reaction process. The SEM, XRD, and FT–IR of 5WO_3_/BiOBr were carried out to further analyze the stability of 5WO_3_/BiOBr (Figures S7 and S8). Figure S7 shows that no obvious change can be found in the XRD patterns and FT–IR spectra of 5WO_3_/BiOBr before and after the reaction, indicating that the structure of the catalyst was stable after the photocatalytic reaction. In addition, the morphology of 5WO_3_/BiOBr did not change before or after the reaction (Figure S8). The above results show that 5WO_3_/BiOBr is stable for photocatalytic CO_2_ reduction.

### 3.6. In Situ FTIR Spectra

**Figure 7:**
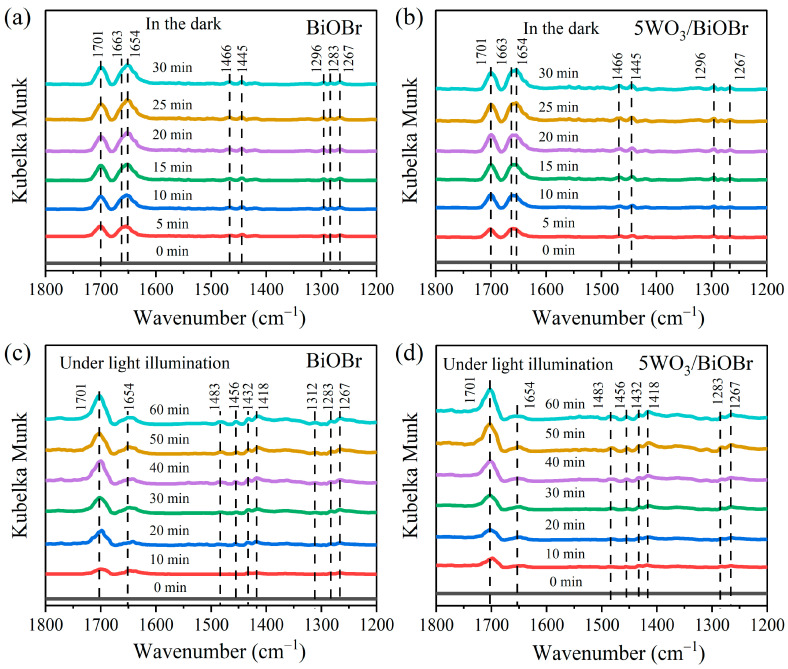
In situ DRIFTS spectra of CO_2_ and H_2_O adsorption on BiOBr (**a**) and 5WO_3_/BiOBr (**b**) in the dark. In situ DRIFTS spectra of CO_2_ and H_2_O adsorption on BiOBr (**c**) and 5WO_3_/BiOBr (**d**) under light irradiation.

In situ DRIFTS spectra were used to investigate the photocatalytic CO_2_ reduction reaction over 5WO_3_/BiOBr (Figure 7). Figure 7a,b show the in situ DRIFTS spectra of CO_2_ and H_2_O adsorption on BiOBr and 5WO_3_/BiOBr in the dark. The peaks at 1663 cm^−1^ and 1654 cm^−1^ are the signals of the water adsorbed on the catalyst surface [62]. The peaks at 1267, 1445, and 1466 cm^−1^ are ascribed to bicarbonate (HCO_3_^−^) [63], the peaks at 1296 and 1312 cm^−1^ are attributed to the monolithic carbonate group (m-CO_3_^2−^) [64], and the peaks at 1267 and 1363 cm^−1^ are assigned to bidentate carbonate (b-CO_3_^2−^) [65]. In addition, the peak at 1701 cm^−1^ was ascribed to COOH^−^. In general, COOH^−^ is the core intermediate for the generation of CO and CH_4_, and its formation time is a critical step for photocatalytic CO_2_ reduction to CO [66].

Figure 7c,d show the in situ DRIFTS spectra of CO_2_ and H_2_O adsorption on BiOBr and 5WO_3_/BiOBr under visible light irradiation. As shown in Figure 7c,d, the characteristic peaks at 1418, 1432, and 1456 cm^−1^ are attributed to HCO_3_^−^, and the peaks in 1483 cm^−1^ are attributed to m-CO_3_^2−^, indicating that new carbon species can be formed under light irradiation. Moreover, the concentration of the CO_2_ intermediates were improved with the increase of the irradiation time. Notably, the concentration of COOH^−^ was significantly improved under light irradiation, which is conducive to the photocatalytic CO_2_ reduction reaction. 

### 3.7. Possible Photocatalytic Mechanism

**Figure 8:**
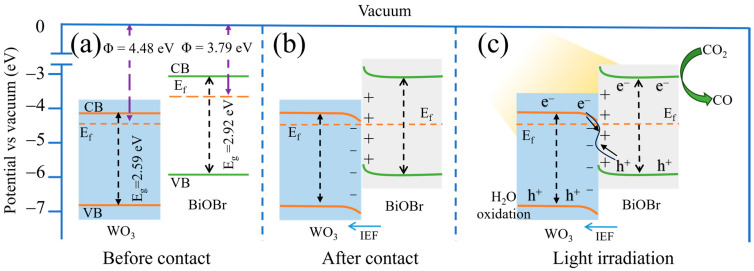
Band energy positions of WO_3_ and BiOBr before (**a**) and after (**b**) contact, S-scheme charge transfer mechanism in WO_3_/BiOBr composites under light irradiation (**c**).

Based on the above discussion, the reaction mechanism of photocatalytic CO_2_ reduction over xWO_3_/BiOBr composites was proposed. As displayed in Figure 8a, BiOBr acts as the reducing photocatalyst, while WO_3_ is an oxidizing photocatalyst, and the Fermi level of BiOBr is higher than that of WO_3_. When BiOBr and WO_3_ are in contact with each other, electrons spontaneously transfer from BiOBr to WO_3_ until the Fermi level reaches equilibrium. In addition, at the interface, BiOBr and WO_3_ are positively and negatively charged, respectively. An electron depletion region is formed at the BiOBr interface and the energy band bends upward. To the contrary, an electron accumulation zone is formed at the WO_3_ interface and the energy band bends downward. In this case, an internal electric field is formed at the WO_3_/BiOBr interfaces (Figure 8b). Under light irradiation, the electrons in the BiOBr and WO_3_ valence bands are excited and then jump to their conduction band. Subsequently, the electrons accumulated in the CB of WO_3_ combine with the holes in the VB of BiOBr (Figure 8c), which follow the S-scheme charge transfer mechanism. In addition, the electrons accumulated in the CB of BiOBr can participate in the photocatalytic CO_2_ reduction, while the holes in the VB of WO_3_ can trigger the H_2_O oxidation. That is, the S-scheme charge transfer mechanism not only separates photogenerated electron-holes efficiently and quickly, but also maintains the strong redox ability of WO_3_/BiOBr composites, which enhances the photocatalytic CO_2_ performance.

## 4. Conclusions

In summary, WO_3_/BiOBr S-scheme heterojunctions were synthesized for photocatalytic CO_2_ reduction. The optimized WO_3_/BiOBr heterostructures exhibited enhanced photocatalytic CO_2_ reduction performance without any sacrificial reagents, and the CO yield reached 17.14 μmol g^−1^ after reaction for 4 h, which was 1.56 times greater than that of BiOBr. The photocatalytic stability of WO_3_/BiOBr was also improved. The enhanced photocatalytic performance can be attributed to the S-scheme charge transfer mechanism, which effectively improves the separation efficiency of photogenerated charge carriers, thus promoting the photocatalytic CO_2_ reduction. This study provides new insights into the construction of efficient and stable S-scheme heterojunction photocatalysts for photocatalytic CO_2_ reduction. 

## Data Availability

The original contributions presented in the study are included in the article/Supplementary Material, further inquiries can be directed to the corresponding author.

## Supplementary Materials

The following supporting information can be downloaded at: https://www.mdpi.com/article/10.3390/ma17133199/s1, Figure S1: Enlarged regions from 20° to 30° in XRD patterns of WO_3_, BiOBr (a), and xWO_3_/BiOBr (b). Figure S2: SEM images of 3WO_3_/BiOBr (a) and 10WO_3_/BiOBr (b). Figure S3: EDX spectrum of 5WO_3_/BiOBr. Figure S4: XPS survey spectra (a) and enlarged spectral regions from 0 eV to 100 eV (b) of BiOBr, WO_3_, and 5WO_3_/BiOBr. Figure S5: UPS spectra of WO_3_ (a) and BiOBr (b). Figure S6: Mott-Schottky plots of BiOBr (a) and WO_3_ (b) at the frequencies of 1000 Hz, 1500 Hz, and 2000 Hz. Figure S7: XRD patterns (a) and FT-IR spectra (b) of 5WO_3_/BiOBr before and after reaction. Figure S8: SEM images of 5WO_3_/BiOBr before (a) and after (b) reaction. Table S1: Specific surface area and average pore size of the samples. Table S2: Comparison of photocatalytic CO_2_ reduction performance over the BiOBr-based photocatalysts. References [45,55,66,67,68,69,70] are cited in the Supplementary Materials.
