# Supplementary material for: WO3/BiOBr S-Scheme Heterojunction Photocatalyst for Enhanced Photocatalytic CO2 Reduction
Source: Materials (Basel). 2024 Jun 30;17(13):3199. doi: 10.3390/ma17133199 (PMC11242261; doi:10.3390/ma17133199)
Supplement: Supplementary file 1 [file materials-17-03199-s001.zip › materials-3044520-supplementary.pdf]

Supporting Information

WO_3/BiOBr S-scheme heterojunction photocatalyst for enhanced photocatalytic CO_2 reduction

Chen Li[1], Xingyu Lu[1], Liuyun Chen[1], Xinling Xie[1], Zuzeng Qin[1], Hongbing Ji[1,2], Tongming Su[1*]

1. Guangxi Key Laboratory of Petrochemical Resource Processing and Process Intensification Technology, School of Chemistry and Chemical Engineering, Guangxi University, Nanning 530004, P. R. China
2. State Key Laboratory Breeding Base of Green-Chemical Synthesis Technology, Institute of Green Petroleum Processing and Light Hydrocarbon Conversion, College of Chemical Engineering, Zhejiang University of Technology, Hangzhou 310014, P. R. China

*Corresponding author:

E-mail: sutm@gxu.edu.cn (T. Su)

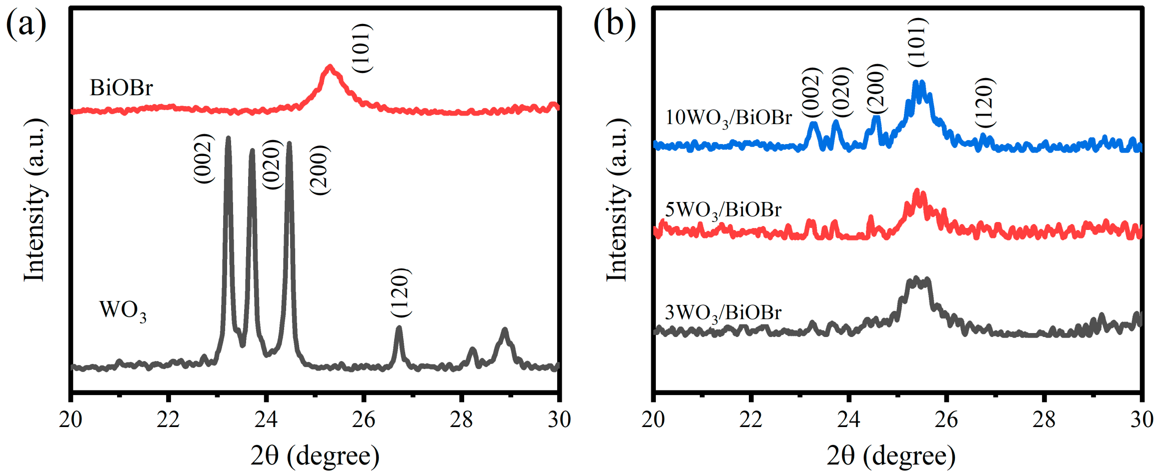

Figure S1. Enlarged regions from 20º to 30º in XRD patterns of WO_3, BiOBr (a), and $xWO_3/BiOBr$ (b).

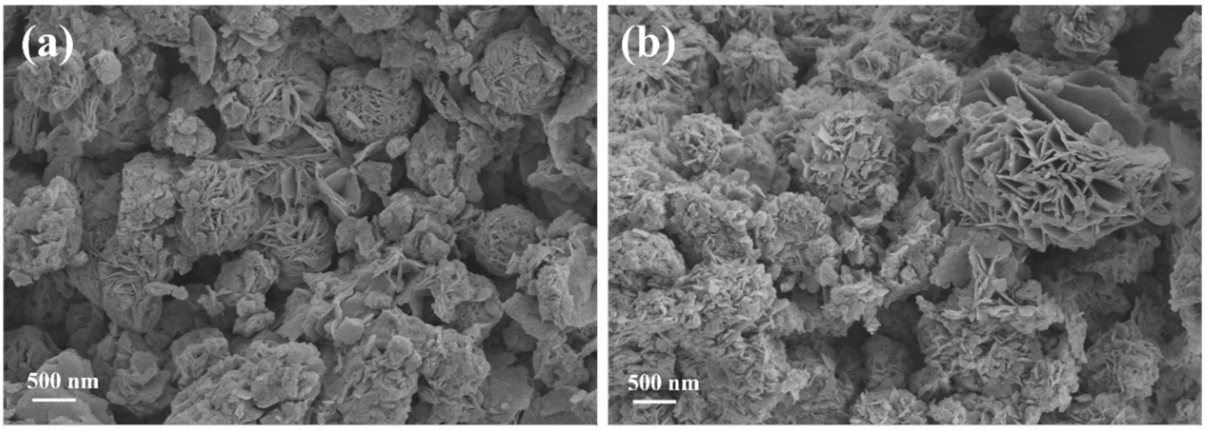

Figure S2. SEM images of $3WO_3/BiOBr$ (a) and $10WO_3/BiOBr$ (b).

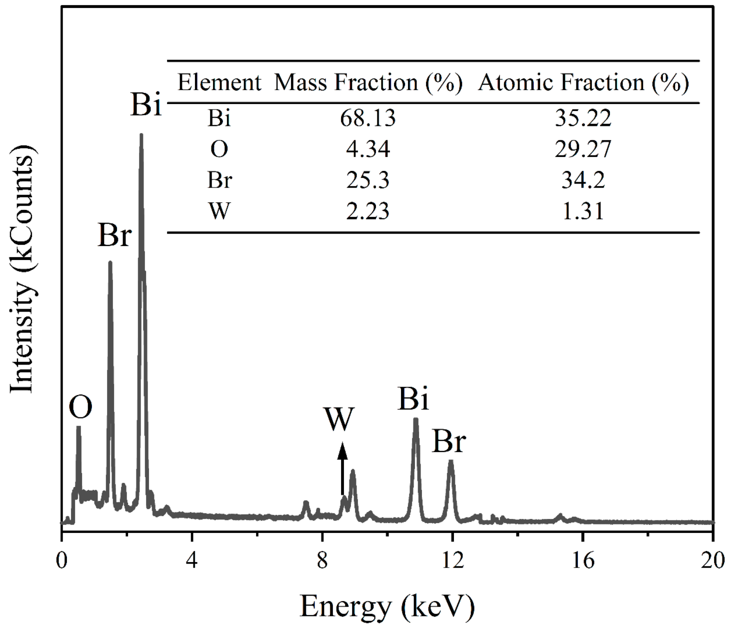

Figure S3. EDX spectrum of $5WO_3/BiOBr$.

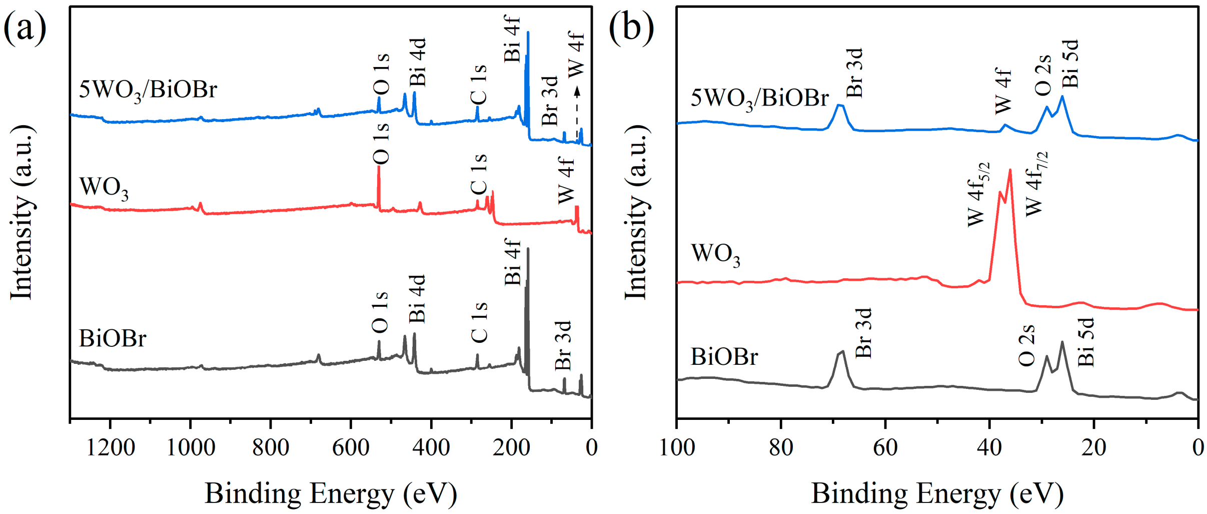

Figure S4. XPS survey spectra (a) and enlarged spectral regions from 0 eV to 100 eV (b) of $BiOBr$, WO_3, and $5WO_3/BiOBr$.

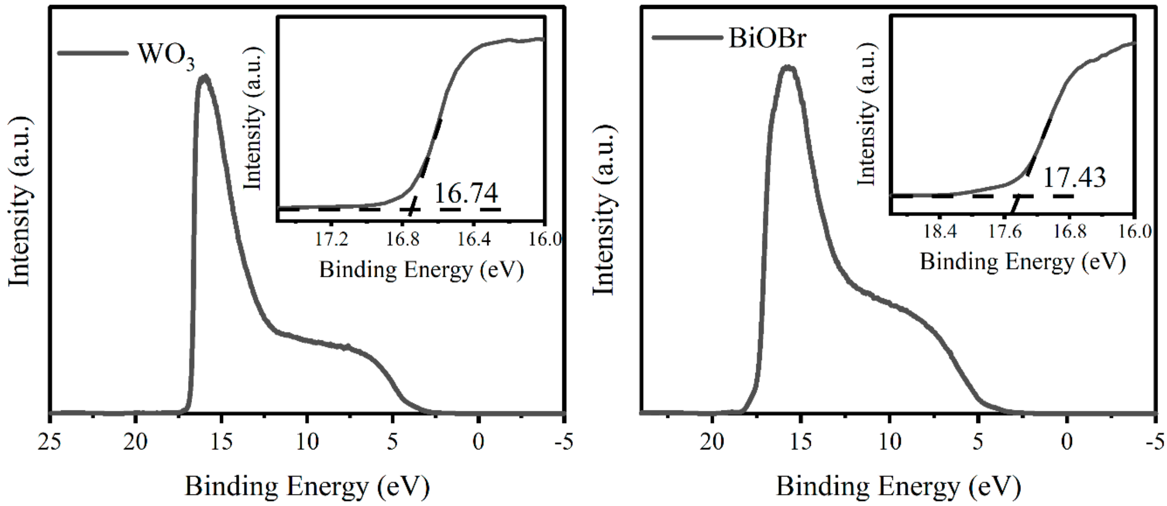

Figure S5. UPS spectra of WO_3 (a) and BiOBr (b).

The work function in the UPS spectra can be calculated by Equations S1:

$$\Phi = 21.22 - E_{cutoff} \qquad S1$$

Where, Φ is the work function, E_{cutoff} is the secondary electron cutoff.

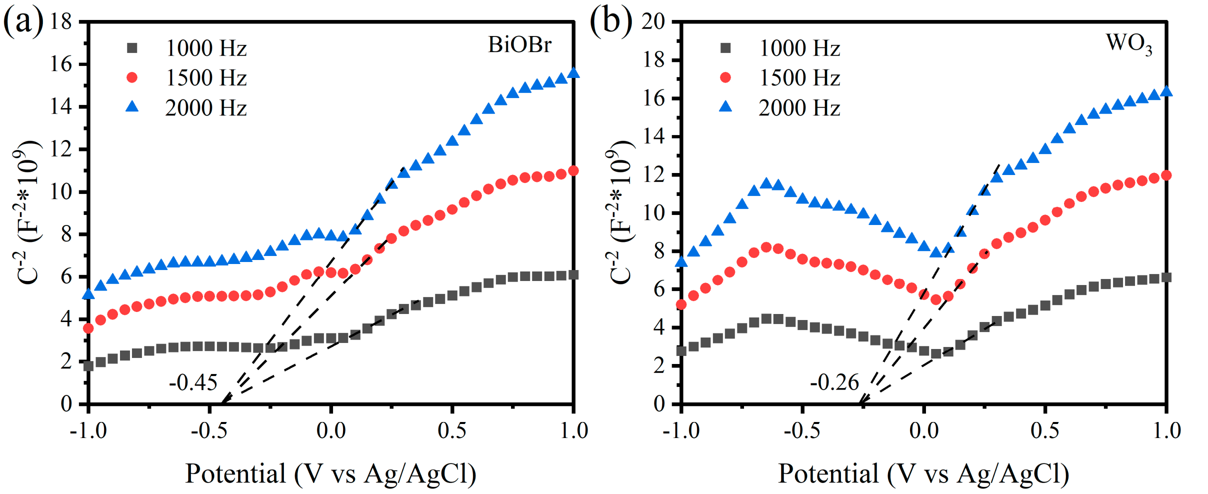

Figure S6. Mott-Schottky plots of BiOBr (a) and WO_3 (b) at the frequencies of 1000 Hz, 1500 Hz, and 2000 Hz.

The potential vs. Ag/AgCl can be converted to potential vs. NHE according to Equations S2:

$$E(\text{NHE}) = E(\text{Ag/AgCl}) + E^{\theta}(\text{Ag/AgCl}) + 0.059\text{pH} \quad S2$$

Where, E^{θ}(Ag/AgCl) = 0.197 V at 25 ℃, pH = 7.

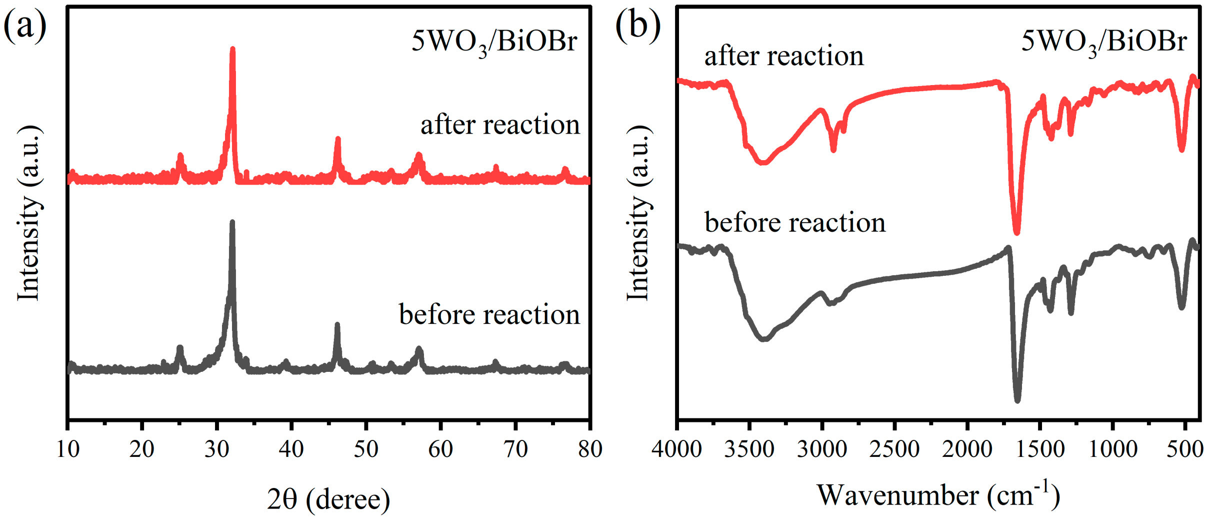

Figure S7. XRD patterns (a) and FT-IR spectra (b) of $5WO_3$/BiOBr before and after reaction.

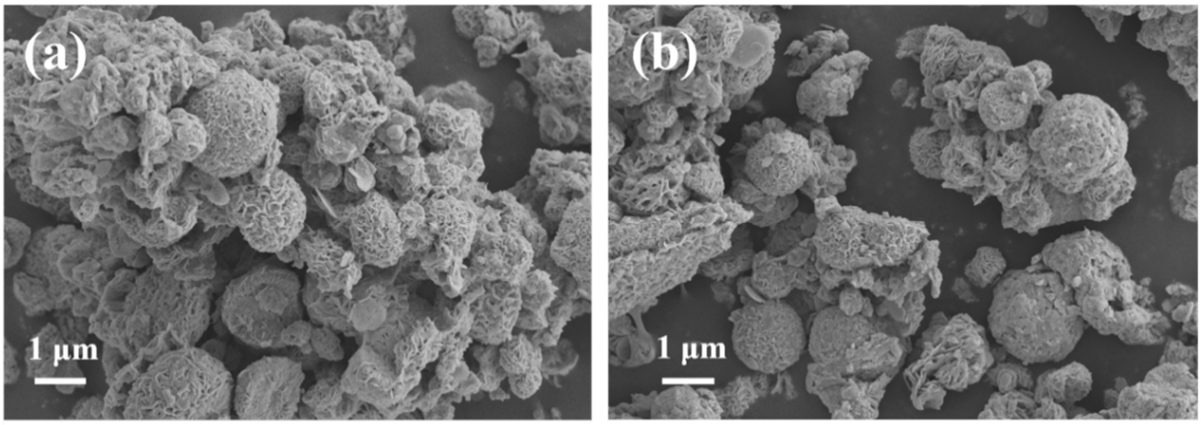

Figure S8. SEM images of $5WO_3$/BiOBr before (a) and after (b) reaction.

Table S1. Specific surface area and average pore size of the samples

Photocatalysts	Specific surface area (m^2 g^{-1})	Average pore size (nm)
BiOBr	19.64	7.84
WO_3	3.54	19.73
$3WO_3$/BiOBr	14.81	7.45
$5WO_3$/BiOBr	17.96	8.05
$10WO_3$/BiOBr	17.15	7.67

Table S2. Comparison of photocatalytic CO_2 reduction performance over the BiOBr-based photocatalysts

Photocatalysts	Light source	CO (μmol g^{-1} h^{-1})	CH_4 (μmol g^{-1} h^{-1})	Refs.
OVs-BiOBr	300 W Xe lamp (λ ≥ 400 nm)	2.03	0	[1]
P/Bi-BiOBr	300 W Xe lamp (λ ≥ 400 nm)	3.1	0.5	[2]
$Zn(OH)_2$/BiOBr	300 W Xe lamp (λ ≥ 420 nm)	5.40	0	[3]
Au-BiOBr	500 W Xe lamp (λ ≥ 420 nm)	2.56	0	[4]
Ag-BiOBr	300 W Xe lamp (λ ≥ 400 nm)	1.37	0	[5]
Zn/BiOBr	300 W Xe lamp (λ ≥ 420 nm)	1.05	2.71	[6]
OVs-BiOBr	500 W Xe lamp (λ ≥ 400 nm)	0	0.49	[7]
WO_3/BiOBr	300 W Xe lamp (λ ≥ 400 nm)	4.29	0	This work

References

1. Lin, W.; Gaopeng, L.; Bin, W.; Xin, C.; Chongtai, W.; Zixia, L.; Jiexiang, X.; Huaming, L. Oxygen Vacancies Engineering ‐ Mediated BiOBr Atomic Layers for Boosting Visible Light ‐ Driven Photocatalytic CO_2 Reduction. *Solar Rrl* **2020**, *5*, 2000480.
2. Jiayu, Z.; Yupei, L.; Xiaojing, W.; Jun, Z.; Yinsu, W.; Fatang, L. Simultaneous Phosphorylation and Bi Modification of BiOBr for Promoting Photocatalytic CO_2 Reduction. *Acs Sustain Chem Eng* **2019**, *7*, 14953-14961.
3. Wenzhen, Q.; Qi, Y.; Hao, Y.; Yu, X.; Zhen, S.; Yue, G.; Yonggui, D.; Yun, L.; Jian, Y.; Geng, L.; Nadeem, R.; Waseem, R.; Jie, Z. Novel 2D/2D BiOBr/$Zn(OH)_2$ photocatalysts for efficient photoreduction CO_2. *Sep Purif Technol* **2023**, *306*, 122721.
4. Xin, F.; ChuYa, W.; Longpeng, Z.; Qi, Z.; Hengdeng, Z.; Qi, W.; Guangcan, Z. Efficient visible-light-driven CO_2 reduction mediated by novel Au-doped BiOBr nanosheets. *Journal of Environmental Chemical Engineering* **2023**, *11*, 109986.
5. Gaopeng, L.; Lin, W.; Bin, W.; Xingwang, Z.; Jinman, Y.; Pengjun, L.; Wenshuai, Z.; Ziran, C.; Jiexiang, X. Synchronous activation of Ag nanoparticles and BiOBr for boosting solar-driven CO_2 reduction. *Chinese Chem Lett* **2023**, *34*, 107962.
6. Guan, C.; Hou, T.; Nie, W.; Zhang, Q.; Duan, L.; Zhao, X. Enhanced photocatalytic reduction of CO_2 on BiOBr under synergistic effect of Zn doping and induced oxygen vacancy generation. *J Colloid Interface Sci* **2023**, *633*, 177-188.
7. Ying, K.X.; Cathie, L.W.P.; WeeJun, O.; SiangPiao, C.; Rahman, M.A. Oxygen ‐ Deficient BiOBr as a Highly Stable Photocatalyst for Efficient CO_2 Reduction into Renewable Carbon ‐ Neutral Fuels. *ChemCatChem* **2016**, *8*, 3074-3081.
